# Information Framing Effect on Public’s Intention to Receive the COVID-19 Vaccination in China

**DOI:** 10.3390/vaccines9090995

**Published:** 2021-09-07

**Authors:** Lihong Peng, Yi Guo, Dehua Hu

**Affiliations:** Department of Biomedical Information, School of Life Sciences, Central South University, Changsha 410013, China; penglh@csu.edu.cn (L.P.); yiguo@csu.edu.cn (Y.G.)

**Keywords:** COVID-19 vaccine, information framing, framing effect, risk disclosure, perceived vaccine efficacy

## Abstract

The aims of the study were (1) to explore information framing effect on the public’s intention to receive the COVID-19 vaccination and (2) to understand the key factors influencing the intention of COVID-19 vaccinations in China. An online questionnaire survey was conducted to explore the influence of demographic characteristics, individual awareness, social relationship, risk disclosure, perceived vaccine efficacy, and protection duration under the assumptions of information framing. The results showed that (1) the persuasion effect under loss frame was higher than that under gain frame (B = 0.616 vs. 0.552); (2) there was no significant difference between sex, age, income, occupation, educational background and residence for the participants’ intention to be vaccinated; whether family members/friends were vaccinated had a strong correlation with their vaccination intention under the gain frame; (3) the higher the understanding of COVID-19 and the compliance with government COVID-19 prevention and control measures were, the higher the vaccination intention was; (4) risk disclosure had the greatest impact on people‘s COVID-19 vaccination intention; (5) perceived vaccine effectiveness and duration of protection had little effect on people’s intention to receive vaccination. The influence of information framing on the intention of COVID-19 vaccination is different. The publicity of relevant health information should pay attention to the influence of information framing and contents on the behavior of public vaccination, so as to enhance public health awareness and promote the vaccination of the whole population.

## 1. Introduction

The COVID-19 pandemic has severely impacted people’s daily life and the normal operation of society. At present, five COVID-19 vaccines have been approved for use in China. As of 31 May 2021, 31 provinces (autonomous regions and municipalities directly under the central government) and Xinjiang Production and Construction Corps in China, reported a total of 661.468 million doses of COVID-19 vaccine. Studies show that only if the rate of COVID-19 vaccination in the population reaches 67%, will the incidence of infection in COVID-19 decline [1]. Therefore, it is very important to improve the vaccination rate of the COVID-19 vaccine.

### 1.1. Information Framing

Individual risk preference usually depends on the expression of the issue [2]. The information framing expresses different health behaviors in two ways: the benefits of taking action—namely, a gain-framed message (e.g., wearing a mask can effectively block the spread of the virus through saliva), and the costs of refusing to take action—namely, a loss-framed message (e.g., without a mask, you are likely to contract COVID-19) [3]. The loss-framed message in disease detection behavior is more convincing, while the gain-framed message in disease prevention behavior is more convincing [3]. There is also a study indicating that a gain-framed message is more persuasive when information is designed to promote a type of behavior, and a loss-framed message is more persuasive when information is designed to prevent a phenomenon [4]. It has been found that in areas that favor prevention, such as using sunscreen cream to prevent skin cancer [5,6] and smoking cessation [7,8], the gain-framed message is more effective than its loss-framed counterpart. Vaccination is a disease prevention behavior. In the preventive behavior of vaccination, the gain-framed message has a stronger persuasion effect than the loss-framed one, that is, the gain-framed message has a stronger influence on people’s vaccination intention [9].

However, people will also be affected when the degree of risk to adopt/reject an act is different: the persuasion effect of the gain-framed message is more strongly perceived when at low risk; the effect of the loss-framed message is more strongly perceived when at high risk [10]. Compared with general preventive behaviors (wearing sunscreen, quitting smoking, etc.), vaccination is at a higher risk, causing a series of reactions such as redness, allergy, even cerebral thrombosis and stroke, thus endangering life. Vaccination is a high-risk behavior [11,12]. Therefore, it is both a preventive and high-risk behavior. Therefore, which frame information has a more forceful persuasion effect on vaccination behavior needs further confirmation.

In the field of studying intention to be vaccinated, a study on the impact of information framing on acceptance of human papillomavirus (HPV) vaccines suggests that the loss-framed message is more likely to increase the intention to receive HPV vaccines than the gain-framed message [13]. Another study found that the loss-framed message increased the intention to receive the MMR vaccine [14]. In addition, studies have explored the impact of information framing on vaccination rates in adults with medical insurance, finding that neither the gain-framed message nor the loss-framed message can improve vaccination rates [15]. There is no consensus on which information framing is most effective in encouraging vaccination. In COVID-19 vaccination, the influence of information framing on vaccination intention has not been studied.

### 1.2. Individual Awareness and Social Relationship

Individual awareness is a psychological function involving the storage, selection, organization, and action plan of information [16]. People with higher health awareness can manage preventive behavior more effectively [17]. Individual awareness here refers to people’s understanding of COVID-19 and their awareness of observing the government’s epidemic prevention and control measures. A study on adult pertussis vaccination of Korean women of childbearing age assessed their knowledge, attitude, and acceptance of pertussis vaccine. The results showed that most women of childbearing age had neither knowledge of pertussis vaccination nor been fully informed of vaccination, resulting in low acceptance [18]. The research on the influence of knowledge of human papillomavirus and vaccination intention shows that people have a less comprehensive understanding of human papillomavirus and have a low understanding of the benefits of vaccination, resulting in a low vaccination rate [19,20,21]. In a study on Chinese college students’ awareness, knowledge, attitude, and intention of HPV vaccination, participants showed high awareness (71% had heard of HPV), but their knowledge about human papillomavirus was limited, which led them to think that the risk of infection was very low, resulting in low vaccination rate [22]. There are surveys on the influencing factors of vaccination in COVID-19 mentioned that people should have higher general vaccine knowledge and their understanding of COVID-19 [23,24,25], but few studies pay attention to the influence of people’s awareness of compliance with government epidemic prevention and control measures on their intention to be vaccinated.

In addition to personal views, in a study on Australians’ willingness to receive the COVID-19 vaccine, 78% received support from their families and friends to receive vaccination, and 84% thought that they should follow the government’s guidelines on vaccines in order to protect community health [26]. Another study reveals that whether an individual’s loved ones/family/friends or a public figure, whom they trust and respect, was vaccinated would affect the person’s willingness to be vaccinated [27]. It can be seen that acquaintance’s vaccination can affect our intention to receive vaccination. However, what will happen if the vaccination rate of acquaintances is low? How the acquaintances’ rate at different levels of framing assumptions will affect people’s intention to be vaccinated? This question needs to be further explored.

### 1.3. Risk Disclosure

In the area of health transmission, risk disclosure refers to the decision to accept or refuse medical intervention or take medication by providing patients with important risk information [28]. For example, prescription drugs require disclosure of the side effects of the drug and the health risks that may arise after use. Research on risk disclosure in healthy behavior has included asking doctors about prescription drugs [29], asking about skin cancer lotion, buying and using healthy products [30], etc. However, few studies have revealed the side effects of the vaccine when investigating vaccine intention.

There is a study on risk disclosure and influenza vaccine, which adds risk disclosure information to the gain-framed message [31], but this research does not discuss the influence of different levels of risk disclosure on people’s intention to become vaccinated under both frames.

### 1.4. Perceived Vaccine Effectiveness and Duration of Protection

Perceived vaccine effectiveness refers to “how effective the vaccine is” and is the main consideration for individual vaccination [32]. A study of vaccination behavior has shown that the information framing and vaccination intention are regulated by sensing vaccine effectiveness [11], which shows that target frame information makes people believe in more effectiveness of the vaccine, thus increasing their vaccination intention. Two studies on H1N1 influenza vaccines have also shown that those who are more convinced that H1N1 vaccines protect them have a higher level of vaccination intention [33,34]. The level of perceived vaccine effectiveness will affect the level of vaccine intention. However, it has not been discussed in the vaccination in COVID-19. There is also no research that explores the impact of vaccine effectiveness on people’s intention to be vaccinated under different assumptions of vaccine efficacy.

A study about parental acceptability of COVID-19 vaccination for children shows that longer protection duration of vaccine-associated with higher parental acceptability of COVID-19 vaccination [35]. The uncertainty of the duration of protection has caused the hesitation of healthcare workers to be vaccinated [36]. How long the duration of protection is acceptable needs to be further explored.

This research is based on the initial stage of COVID-19 vaccination in China, using the online questionnaire platform to distribute the questionnaire. The survey was carried out for one month to explore the influence of information framing on COVID-19 vaccination behavior and provide a scientific basis for the intervention plan to improve vaccination rate, so as to provide a reference for prevention and control measures such as vaccination of related diseases during and after the epidemic.

## 2. Materials and Methods

### 2.1. Investigation Method

This study explored the influence of information framing on people’s intention to COVID-19 vaccination. Ethical approval to conduct the study was obtained from the Institutional Review Board of College of Life Sciences, Central South University (Reference No.: 2020-1-44), and conducted following the guidelines of the Declaration of Helsinki. Two different kinds of questionnaires (loss frame (Volume A) and gain frame (Volume B)) were distributed by an online questionnaire platform. The data collection process was led by all research team members, and the survey link was advertised and distributed to the research team members via social software such as WeChat, QQ. Further, members of the network were requested to spread the survey. The participants received one of the questionnaires at random. The questionnaire indicated that if you have already filled in volume A, please do not fill in Volume B, excluding the possibility of cross filling. The questionnaire was distributed from 31 March 2021to 30 April 2021, totaling one month, the initial stage of COVID-19 vaccination in China. Inclusion criteria were (1) not vaccinated with COVID-19 vaccine and (2) informed consent and voluntary participation in this study. Exclusion criteria were (1) having a mental illness or cognitive impairment and (2) refusing to participate in this study.

Prior to the survey, a pilot study was conducted among 30 subjects to ensure that there were no problems in reading the frame information, understanding and answering the questions in the questionnaire. Most participants said the frames were easy to understand and the length of the questionnaire was appropriate.

Finally, 280 valid questionnaires were obtained by excluding invalid questionnaires. The data collected were analyzed by SPSS 21.0 and SmartPLS 3.0, and the statistical significance was defined as *p* < 0.05 (double tail). The Cronbach’s alpha values of the two groups were 0.918 and 0.904, respectively, both of which were greater than 0.7, which proved that the questionnaire had good reliability, and the KMO values were 0.843 and 0.848, respectively, both of which were greater than 0.6, which proved that the questionnaire had good validity and could be formally analyzed.

### 2.2. Investigation Content

The questionnaire began with an introduction to the purpose of the study, a brief introduction to COVID-19, and progress in vaccine development. The questionnaire consisted of three main sections. The first section was the subjects’ basic information, such as sex, age, income, occupation, place of residence, educational level, the understanding of COVID-19, and compliance with government COVID-19 control measures. In the second part, participants were asked to read four stimulus messages, all of which were designed according to the characteristics of the gain and loss frames. After reading the stimulus information, the subjects continued to respond to the Intention to become vaccinated. In the third part, participants continued to read questions about the vaccine’s effectiveness, side effects, duration of protection, and vaccination rate of acquaintances, then to answer the intention to be vaccinated.

### 2.3. Information Framing and COVID-19 Vaccine

All the information involved in this study was designed according to the characteristics of the gain and loss frames based on the literature review, resident interviews, and expert consultation. In addition, to avoid the impact of the amount of information, the number of words between two frames is similar and controlled at about 250. Stimulation information is shown in Table 1.

### 2.4. Variable Measurement

This study involved the measurement of vaccination intention to COVID-19 and the measurement of it under various framing scenarios, all of which used the Likert 5 scale. The higher the value was, the higher the vaccination intention to COVID-19 was. All the measurements of related factors were adapted from existing research, and the contents were determined after interviews with experts as follows: (1) The vaccination intention to COVID-19 was adapted from Fishbein and Ajzen [37], “Your vaccination intention to COVID-19 is?”; “If the vaccination of COVID-19 is free, how likely are you to be vaccinated?”; “If you need to make a queue for vaccination to COVID-19, how likely are you to be vaccinated?”; (2) the measurement of vaccination intention under different framing information was adapted from Yu et al. [38]; four specific situation combinations were measured, mainly including vaccine safety, effectiveness, duration of protection, and acquaintance vaccination rate, such as “If COVID-19 vaccine is 60% effective, how likely are you to be vaccinated”; “If COVID-19 vaccine will cause side effects such as swelling and pain at the vaccination site, general fever, fatigue, etc., how likely is your vaccination”; “If the protection duration of the vaccine is 2 years, how likely are you to be vaccinated”, etc.; (3) according to the existing research foundation, there were 9 control variables involved in this study: gender, age, income, occupation, place of residence, education level, whether family/friends are vaccinated, the degree of understanding of COVID-19, and the degree of compliance with the government’s COVID-19 prevention and control measures.

## 3. Results

### 3.1. Effect of Demographic Characteristics on Vaccination Intention Based on Information Framing

A total of 280 valid questionnaires were collected. Two variables (sex and place of residence) were tested by independent sample *t*-test, and four variables (age, income, occupation, and educational background) were analyzed by variance (ANOVA).

According to Table 2, men’s intention to be vaccinated is higher than that of women under different frames. The intention of male subjects to receive vaccination increased when they received loss-framed messages, but there was no significant difference between male and female subjects when receiving gain-framed messages. In addition, an independent sample *t*-test showed a non-significant trend between sex and place of residence on the intention to receive vaccination. Through the analysis of variance (ANOVA), age, income, occupation, educational background did not reach a significant level of intention to be vaccinated, indicating that these demographic characteristics did not significantly change the intention to be vaccinated. In addition, the intention of undergraduate and below groups is higher than that of graduate students and above.

### 3.2. Effect of Individual Awareness and Social Relationship on Vaccination Intention

#### 3.2.1. Effect of Individual Awareness on Vaccination Intention

According to Table 3, through the analysis of variance (ANOVA), we found that under the gain frame, the degree of understanding of COVID-19 (*p* = 0.013 < 0.05) and compliance with the government COVID-19 control measures (*p* = 0.000 < 0.05) had a significant correlation with intention. Under the loss frame, the degree of understanding of COVID-19 (*p* = 0.000 < 0.05) and the degree of compliance with the government COVID-19 control measures (*p* = 0.007 < 0.05) were also significant. This means the higher understanding of COVID-19 leads to more compliance with government COVID-19 prevention and control measures and higher vaccination intention.

#### 3.2.2. Effect of Social Relationship on Vaccination Intention

According to the data in Table 4, the vaccination rate of acquaintances is significantly correlated with the intention to be vaccinated under all information framing assumptions (*p* < 0.001). After receiving the information about the acquaintances’ vaccination rate, the intention of the subjects decreased in the first two degrees (30%,60%) and increased slightly in the third degree (90%). Even if 60% of acquaintances around them are vaccinated, people still hold a wait-and-see attitude. When 90% of acquaintances were vaccinated, they only increased their intention to be vaccinated slightly more than without the assumption (M = 4.24 vs. M = 4.38; M = 4.16 vs. M = 4.38). Studies show that only if the vaccination rate in the population reaches 67%, will the incidence of infection decrease. It can be seen that 60% of the acquaintance vaccination rate still cannot effectively persuade people to be vaccinated. Therefore, it is difficult to achieve the immunization rate by people’s conscious vaccination.

According to Table 5, within the gain frame, there was a significant difference in the intention of whether family members/friends to be vaccinated (*p* = 0.001 < 0.05). When family members/friends are vaccinated with COVID-19 vaccination (M = 4.35, SD = 0.655), their willingness to be vaccinated is much higher than that of those who have no family members/friends who are vaccinated (M = 3.87, SD = 0.924). Under the loss-framed information, a nonsignificant trend is observed (*p* = 0.722 > 0.05), indicating that regardless of whether family members/friends are vaccinated, people’s intention to be vaccinated is relatively high.

### 3.3. Effects of Information Framing on Vaccination Intention

The information framing involved in this study was divided into loss-frame and gain-frame groups. As shown in Table 6, the mean intention to receive the COVID-19 vaccine (M = 4.24, SD = 0.866; M = 4.37, SD = 0.766) in the loss-frame group was higher than in the gain-frame group (M = 4.16, SD = 0.807; M = 4.31, SD = 0.774). The information framing had a significant correlation with the vaccination intention to receive the COVID-19 vaccine (*p* < 0.001). However, the comparison between the two frames is not obvious. Through further analysis of linear regression analysis, we found that the absolute value of the standardized beta coefficient under the loss frame is larger than that under the gain frame (B = 0.616 > B = 0.552, *p* < 0.001). This indicates that the loss frame has a greater impact on intention to receive COVID-19 vaccination than the gain frame. In the case of emphasizing the free cost of vaccination, the intention to be vaccinated increases under both frames. The promotion of free vaccination policy in China has increased vaccination coverage to some extent. However, a comparison of the absolute value of the standardized beta coefficient between the loss frame and the gain frame is not obvious (B = 0.765 ≈ B = 0.767, *p* < 0.001).

Overall, the information framing can influence people’s intention to receive COVID-19 vaccination, and the loss frame is more persuasive than the gain frame, but when we emphasize the free cost of vaccination, the comparison of influence to be vaccinated between two frames is minute.

### 3.4. Effect of Risk Disclosure on Vaccination Intention

By using the difference of intention score between the second part and the third part of the questionnaire, we can gain insight into the influence of the information framing on vaccination intention after the participants received the information of other factors. By comparing the gain and loss frame results, the effects of other factors on the gain/loss frame can be obtained.

According to the data in Table 7, after receiving information about the side effects of the COVID-19 vaccine, the subject’s intention changed significantly. If the side effects of vaccines are fever, redness and swelling, people may choose to be vaccinated under the loss frame (0.27, SD = 1.274, *p* < 0.001); however, they tend to wait and see under the gain frame (M = 2.96, SD = 1.084, *p* < 0.001). In addition, when vaccination may lead to cerebral thrombosis, stroke, people tend to wait and see under the loss frame (M = 2.40, SD = 1.343, *p* < 0.001) and likely to refuse under the gain frame (M = 2.00, SD = 1.043, *p* < 0.001). If vaccination affects life safety, people mostly tend to have a refusal attitude (M = 2.04, SD = 1.368, *p* = 0.014; M = 1.69, SD = 1.096, *p* = 0.025). Compared with not disclosing these risks (M = 4.24, SD = 0.866, *p* < 0.001; M = 4.16, SD = 0.807, *p* < 0.001), different levels of risk disclosure have caused significant decline in vaccination intention.

### 3.5. Effect of Perceived Vaccine Effectiveness and Duration of Protection on Vaccination Intention

According to the data in Table 8, perceived vaccine effectiveness is significantly correlated with the intention to be vaccinated under all information framing assumptions (*p* < 0.001). After receiving the information about the effectiveness of the COVID-19 vaccine, the subject’s intention declined in the first two degrees (40%,60%), and increased in the third degree (90%). When the vaccine effectiveness is 40%, people tend to take a wait-and-see attitude (M = 3.43, SD = 1.166, *p* < 0.001; M = 3.09, SD = 1.060, *p* < 0.001); when the vaccine effectiveness is 60%, people may be willing to become vaccinated (M = 3.93, SD = 0.924, *p* < 0.001; M = 3.61, SD = 1.046, *p* < 0.001); when the vaccine effectiveness is 90%, people tend to be very willing to become vaccinated (M = 4.55, SD = 0.854, *p* < 0.001; M = 4.38, SD = 0.900, *p* < 0.001). It shows that when the vaccine effectiveness is close to 90%, the propaganda of vaccine effectiveness can improve people’s vaccination intention (M = 4.24 vs. M = 4.55; M = 4.16 vs. M = 4.38).

According to the data in Table 9, after receiving the information about the duration of protection of the COVID-19 vaccine, the intention of the subjects declined in the first two degrees (half a year, one year), and the third degree (two years) was slightly lower than before receiving the assumed information (M = 4.17 vs. M = 4.24; M = 4.09 vs. M = 4.16). Under the loss frame, when the duration of protection is half a year or one year, people may choose to become vaccinated (M = 3.45, SD = 1.137, *p* < 0.001; M = 3.83, SD = 1.035, *p* < 0.001); when the duration of protection is two years, people are more willing to become vaccinated (M = 4.17, SD = 1.009, *p* < 0.001). Under the gain frame, when the duration of protection is only half a year, people hold a wait-and-see attitude (M = 3.23, SD = 1.059, *p* < 0.001); when it is one year, people may choose to become vaccinated (M = 3.59, SD = 1.068, *p* < 0.001) and more willing to become vaccinated when two years (M = 4.09, SD = 1.014, *p* < 0.001). It shows that the COVID-19 vaccine’s minimum acceptable standard of duration of protection is 2 years or more. People are more inclined to protect themselves and choose to wait and see instead of being vaccinated with a duration of protection of less than 2 years.

## 4. Discussion

### 4.1. Effect of Demographic Characteristics on Vaccination Intention

Under different information frames, men are more likely than women to be vaccinated, which may be associated with a higher risk of COVID-19 complications and death among men [39]. In addition, men have lower trust in rumors and negative information about the COVID-19 vaccine. Then they have a higher awareness of the risk of the disease [40,41]. In addition, this study shows that, under the framing information, COVID-19 vaccination intention is not much related to age, but some studies show that older people are more willing to be vaccinated with the COVID-19 vaccine [42,43]. The reason why our study does not reach a consistent conclusion may be that most subjects are concentrated between 18 and 59 years old, and there are fewer adolescent and elderly groups. One study suggests that high incomes are positively correlated with vaccine intention, as they have less economic pressure to be vaccinated [44,45]. However, our research shows that high-income groups show a lower intention to be vaccinated than other income groups. This may be related to better prevention and control of the epidemic in China, with high-income people having sufficient funds to obtain more protective equipment and better medical conditions. The same lower intention of the highly educated group (master’s degree and above) may be associated with their ability to access and process more information and more consideration of vaccine safety and effectiveness.

Understanding these demographic-based vaccination intentions can inform government and public health authorities about which segments of the population need to be targeted, so as to increase coverage in COVID-19 vaccination.

### 4.2. Effect of Individual Awareness and Social Relationship on Vaccination Intention

#### 4.2.1. Effect of Individual Awareness on Vaccination Intention

Our study suggests that the higher the population’s understanding of COVID-19 is, the higher the intention to receive vaccination is. There is a positive correlation between knowledge, health literacy, and COVID-19 vaccination intention [46,47,48]. This is consistent with our findings. In addition, the higher the compliance with government COVID-19 prevention and control measures is, the higher the intention becomes. Most recipients of COVID-19 vaccines may be those who trust their governments, such as those in South Korea and China [48,49]. Those who have high confidence in their health systems [50] or in the information provided by public health systems [51] are more likely to be vaccinated.

This result has many implications for the public health sector and public education. Firstly, the public health sector needs to highlight the dangers of the new coronavirus, the actual impact of the COVID-19, and the severity of the public health threat; secondly, the benefits of the COVID-19 vaccine can be effectively publicized through authoritative media or spokespersons (such as doctors) to increase public understanding of the vaccine and dispel their negative opinions; finally, educational institutions or the public health sector can also guide the public to view the vaccination behavior and improve the vaccination rate from a positive direction by organizing activities such as popular science lectures on COVID-19 vaccines.

#### 4.2.2. Effect of Social Relationship on Vaccination Intention

With the increase of the vaccination rate of acquaintances around them, the intention of the subjects to be vaccinated would increase. In one study, 78% of respondents said their decision to become vaccinated was supported by family and friends [27]. When people’s family and friends are vaccinated, their intention is stronger [28].

There is a significant correlation between the intention of the participants to receive the vaccine and whether their family members/friends received the vaccine under the gain frame, but there is no consistent result under the loss frame. This may be related to the prevention and control of the COVID-19 epidemic in China. Long-term pandemics and low mortality rates may lead to fatigue in implementing preventive measures, making people too optimistic about the risk of disease and less fearful, and some people may hesitate to take the COVID-19 vaccine [52]. Under the loss frame, people think that vaccination should be carried out even from the perspective of their own risks. Therefore, regardless of whether family members/friends are vaccinated or not, their willingness to be vaccinated is high. However, the gain-framed information suggests that if we become vaccinated, we will achieve group immunization, and even impact on opening the door to a societal immunization. In the case of affecting society and even the country, they are likely to be persuaded to be vaccinated in order not to affect the collective security and interests. The attitudes and beliefs associated with vaccination include the expected benefits of vaccinating yourself and the community with the COVID-19 vaccine, and the perceived community benefits of vaccination are positively correlated with vaccination intention [52]. It can be seen that emphasizing collective interests for those who are unwilling to become vaccinated may encourage them otherwise. Therefore, for a subset of people who have a low intention to be vaccinated, priority can be given to persuading their family members/friends to be vaccinated or to those more influential in the community to take the lead [28] and enhance their intention to be vaccinated.

### 4.3. Effect of Information Framing on Vaccination Intention

To some extent, the results of the gain frame may reflect that it is difficult to believe the COVID-19 vaccine will produce immediate effects or long-term benefits. Therefore, it has little impact on vaccination intention. Part of the reason is the belief that even if a COVID-19 vaccine is administered, there is still a risk of infection due to a low population vaccination rate; moreover, the vaccine is currently rapidly developed, the time to market is short, and the effectiveness and duration of protection have not been strictly confirmed, so the benefits of vaccination may not be obvious.

The results of the loss frame, to a certain extent, suggest that without vaccination, it is believed that the COVID-19 infection could have disastrous consequences in the future, even bringing about a new round of crises for society and the country. In situations where there is a degree of threat to individuals, society, and even the state, people are more likely to be persuaded to take COVID-19 vaccines. Overall, the impact of loss frame on vaccination intention is higher than that of the gain frame. 

When we emphasize that vaccination is free, the willingness to be vaccinated generally rises. It is worth noting that the impact between the loss frame and the gain frame is minute when the vaccine is free. The possible reason is that people are worried about the consequences of not vaccinating when exposed to the loss frame, and therefore, they will be more willing to become vaccinated regardless of whether they are charged or not. However, when exposed to the gain frame, people do not feel the real crisis, and they may take a wait-and-see attitude if they do not know whether the vaccination is free or not. However, it will inspire them to be vaccinated when vaccination is free [53,54]. Therefore, the impact on the intention to be vaccinated has increased.

To summarize, in order to improve the vaccination rate of the COVID-19 vaccine, on the one hand, the public health department should publicize more authoritative data to show the effectiveness of the vaccine and take some authoritative measures to alleviate people’s concerns about the side effects of the COVID-19 vaccine; on the other hand, it could spread more information about social responsibility and enhance the national anti-epidemic force, urging people to actively take COVID-19 vaccine and complete group immunization at an early date. In addition, the promotion of free vaccines will increase the vaccination rate.

### 4.4. Effect of Risk Disclosure on Vaccination Intention

Risk disclosure does not in itself represent a loss frame, as it does not change the broad context of the gain/loss frame. Instead, it adds a potential health threat that is often considered when vaccinated. Risk is defined in this study as a possible side effect of the vaccine. Foreground theory points out that people tend to risk averse when they are provided with the gain-framed message and to risk seeking when they are provided with the loss-framed message. Foreground theory proposes another form of risk, which is to avoid the risk of illness. That is, when people are exposed to the benefits of COVID-19 vaccination (gain frame), they tend to be vaccinated to avoid disease; when they are exposed to the disadvantages of non-vaccination (loss frame), they tend to opt for vaccination, accepting the side effects of the vaccine, avoiding a greater risk by seeking a smaller risk.

Our findings suggest that individuals are more likely to receive the COVID-19 vaccine when only the benefits of the COVID-19 vaccination are demonstrated without disclosure of possible side effects. The potential risk of the COVID-19 vaccine included in the information significantly reduces the intention to receive vaccination. The intention to be exposed to a gain-framed message is stronger than to be exposed to a loss-framed message. This conclusion is basically consistent with the perspective of prospect theory that people are vaccinated in order to guard against greater risks but tend to deny vaccination when the vaccination behavior itself contains greater risks or even exceeds the risk of refusal. Studies show that those who believe they are at greater risk or risk of infection are more likely to receive vaccines [55,56,57,58]. A survey of India’s intention to vaccinate COVID-19 found that risk disclosure was not significantly associated with India’s intention to become vaccinated [59], where non-vaccination risks are higher than the side effects of the vaccine itself, resulting in inconsistent results with our findings.

Previous studies have shown that risk disclosure has a negative impact on behavioral outcomes [29,31]. Given that the findings of this study demonstrate that risk disclosure has a significant negative impact on vaccine intention, both within the gain and loss frames, this concept may be a significant predictor of vaccine advocacy information strategy. While it is important to disclose the side effects of the vaccine, this behavior may eventually lead to individual rejection in propaganda. Therefore, it may be more effective not to include risk disclosures if the goal of information dissemination is to increase the intention to be vaccinated. However, in practice, it may be considered to disclose risk information while adding as much information as possible to address public concerns and improve vaccination coverage.

### 4.5. Effect of Perceived Vaccine Effectiveness and Duration of Protection on Vaccination Intention

A study of the intention of Hong Kong citizens to receive COVID-19 vaccines found that perceived vaccine efficacy and duration of protection were associated with the intention to become vaccinated [38]; the result is consistent with our findings. Due to the particularity of the COVID-19 epidemic situation, the vaccine was developed in a relatively short period of time. The effect of immunization and the length of the immunization duration were uncertain, which adversely affected the effectiveness of the perceived vaccine [60].

The US Food and Drug Administration has set a minimum acceptable efficacy level of 50% for COVID-19 vaccines [61], which requires very high (>70%) population coverage immunization [62]. A study has shown that even if the vaccine is 80% effective, at least 75% of vaccine coverage is required to control the COVID-19 pandemic [63]. People have a low perception of the effectiveness of newly marketed COVID-19 vaccines but have a high expectation and demand for vaccine effectiveness, leading to a general decline in people’s intention to be vaccinated. Our research shows that the effect of information about vaccine effectiveness is more negative, which may be related to the increase of COVID-19 variants and the severe global epidemic situation. Adding information about vaccine effectiveness in publicity information may not be significant or even have a negative impact on people’s intention to be vaccinated.

Since the vaccine has just been released to the market, the duration of its protection cannot be confirmed. In our offline pilot survey, we found that if the duration of vaccine protection is only half a year or one year, people are more inclined to choose not to be vaccinated, they tend to do a good job of self-protection. In our online survey, when the duration of protection is two years or more, people’s intention to be vaccinated rises. Healthcare professionals expressed the need for trust to be established in the whole vaccination process. Evidence-based information about vaccines should be provided to improve the vaccination rate [64].

After the statistical analysis of the samples in the second and third parts, in the context of different framing information, the risk disclosure has the biggest fluctuation effect on people’s vaccination intention. This result is consistent with the answer (safety) set of “which aspect of vaccine do you care most about” in the last question of the questionnaire (N = 224 (80%)). In addition, the effectiveness and duration of protection of the vaccine may have an impact. Finally, according to the comparison of the results of the intention in the loss/gain frame, the score of each dimension of the loss frame is basically higher than that of the gain frame, and it is proved again that the persuasion effect of the loss frame on vaccination is higher than that of the gain frame.

## 5. Conclusions

Information frames were added to the study on the public’s intention to receive the COVID-19 vaccine under different assumptions such as vaccine effectiveness/risk disclosure/duration of protection/acquaintance vaccination rate in order to provide a scientific basis for the intervention plan to improve the vaccination rate of the COVID-19 vaccine and provide a reference for prevention and control measures such as vaccination of related diseases during and after the epidemic. This study used a questionnaire to analyze the impact of different information frames on people’s intention of COVID-19 vaccination. The main conclusions are as follows:

(1) Information framing has an influence on the intention of COVID-19 vaccination. From the perspective of information itself, the different organizational forms of information have different persuasive effects. Therefore, public health departments, medical institutions, and other departments should pay attention to the use of information frames to promote healthy behavior.

(2) In general, in COVID-19 vaccination, the effect of persuasion under the loss frame is higher than that under the gain frame. However, the impact between the two frames is minute when the vaccine is free. While calling on the public to be active in vaccination, the government can start with a loss frame to achieve better persuasion and promote free vaccination of vaccines.

(3) There was no significant difference between sex, age, income, occupation, educational background, and place of residence in the subjects’ intention to be vaccinated. Whether family members/friends are vaccinated has a strong correlation with vaccination intention under the gain frame. Emphasizing collective interests for those who are unwilling to become vaccinated may encourage them to receive the vaccine, and priority can be given to persuading their family members/friends to be vaccinated against COVID-19.

(4) The higher the understanding of COVID-19 and the compliance with government COVID-19 prevention and control measures are, the higher the vaccination intention is. Public health departments and medical institutions should propagate COVID-19 awareness to the public; meanwhile, the government should reinforce the popularization of prevention and control measures.

(5) If the goal of information dissemination is to increase the intention of vaccination, it may be more effective not to include risk disclosure information. However, in practice, it may be considered to disclose risk information while adding as much information as possible to address public concerns and improve vaccination coverage.

(6) The inclusion of information on effectiveness and duration of protection in promotional information may not be significant in increasing people’s intention to be vaccinated. Perhaps because of the persistence and variability of the global COVID-19 epidemic, most people believe that even if vaccinated, they may not be able to protect against the changing virus.

## 6. Limitations and Prospects

The results of this study provide theoretical and practical insights into the promotion of healthy public behavior. The loss frame is more persuasive than the gain frame on COVID-19 vaccination intention in general. Risk disclosure has a significant negative impact on vaccination intention, and therefore, it may be a meaningful predictor of vaccine advocacy information strategies. However, there are still deficiencies in the study.

On the one hand, a convenience sample was adopted in this study. Although the randomicity of samples is strong, most participants are young and middle-aged groups. The information framing effect on the intention of adolescents and elderly groups to receive the COVID-19 vaccine needs further study.

On the other hand, our study only tests the current vaccination intention of unvaccinated people after reading the framing information; future studies should also measure and compare people’s intention to continue or change after they have a deeper perception of the benefits and side effects of vaccination (e.g., after the first dose of vaccine).

There is still much room for the development of the application of information framing in the field of health. Aside from the frames of gain and loss involved in this study, there are still many forms of research methods to broaden the research and apply information framing in the health sector.

## Figures and Tables

**Table 1:** Stimulation information used in the questionnaire.

Information	Loss-Framed Messages	Gain-Framed Messages
1	Nanshan Zhong, an academician of the Chinese Academy of Engineering and head of a high-level expert group at the National Health Commission, said: “The longer it takes to get vaccinated, the more likely there will be more mutant strains.”	China’s biological COVID-19 inactivated vaccine protection rate is nearly 80%. At present, more than 1 million people have been vaccinated, and no serious adverse reactions have occurred to any of them. None of the tens of thousands of people who have gone overseas to high-risk countries and regions have been infected, which fully proves the safety and effectiveness of the vaccine.
2	Wenhong Zhang stressed: “If the vaccination is not in place, the speed is not fast enough, there is a possibility that the disease will spread again in the future, the population infection rate will increase.”	If you are vaccinated against COVID-19, the risk of infection and the severity of illness in COVID-19 are lower than those of unvaccinated people. Even if infected with COVID-19 virus, the probability and severity of sequela can be reduced.
3	If you are not vaccinated against COVID-19, you are highly likely to have respiratory infections, lung damage, cardiovascular disease, multiple organ dysfunction, etc. After recovery, some patients still have difficulty breathing, fatigue, depression, anxiety, hair loss, brain fog, and other conditions.	“The more people get the vaccine, the faster we get the group immunization, and we can gradually build up the immune barrier in the population and stop the COVID-19 epidemic,” said the Director of China CDC.
4	“The world is expected to reopen from the end of this year or the beginning of next year,” said Dr. Wenhong Zhang. “Vaccines are better hit this year and as soon as possible, as vaccinations may become crowded over time.”	“The time we expect to open our doors does not depend on others, but on our own epidemic management and vaccination,” said Dr. Zhang.

**Table 2:** Effect of demographic characteristics on vaccination intention based on information framing.

Variables		N (%)	Gain Frame			Loss Frame		
			M(SD)	F	*p*	M(SD)	F	*p*
Sex	Male	104(37.1%)	4.19(0.851)	0.323	0.747	4.40(0.799)	1.799	0.074
	Female	176(62.9%)	4.14(0.787)			4.14(0.897)		
Residence	City	215(76.8%)	4.20(0.766)	1.334	0.184	4.18(0.899)	−1.430	0.155
	Rural	65(23.2%)	3.96(0.976)			4.40(0.767)		
Age	≤18	3(1.1%)	4.00(0.000)	0.173	0.952	5.00(0.000)	1.317	0.267
	18–29	164(58.6%)	4.13(0.801)			4.15(0.894)		
	30–49	85(30.3%)	4.21(0.857)			4.33(0.822)		
	50–59	25(8.9%)	4.22(0.833)			4.13(0.957)		
	≥60	3(1.1%)	4.50(0.707)			3.00(0.000)		
Revenue (Yuan)	≤3000	111(39.6%)	4.11(0.831)	1.510	0.203	4.26(0.905)	0.088	0.986
	3001~5000	73(26.1%)	4.11(0.737)			4.24(0.773)		
	5001~10,000	67(23.9%)	4.41(0.682)			4.21(0.935)		
	10,001~20,000	23(8.2%)	4.18(0.874)			4.33(0.888)		
	>20,000	6(2.2%)	3.50(1.291)			4.00(1.414)		
Occupation	Student	91(32.5%)	4.06(0.866)	2.126	0.066	4.28(0.841)	1.840	0.109
	Civil Servants	9(3.2%)	4.80(0.447)			4.75(0.500)		
	Employees of enterprises and institutions	80(28.6%)	4.42(0.732)			3.98(0.976)		
	self-employed individual/Freelancer	51(18.2%)	3.93(0.829)			4.46(0.730)		
	Farmers	13(4.6%)	4.33(1.155)			4.50(0.707)		
	Others	36(12.9%)	3.94(0.556)			4.16(0.898)		
Education	Junior college and below	112(40%)	4.18(0.834)	2.512	0.085	4.34(0.816)	0.885	0.415
	Undergraduate	88(31.4%)	4.31(0.668)			4.15(0.958)		
	Master and above	80(28.6%)	3.96(0.903)			4.14(0.879)		

Note. M = mean; SD = standard deviation.

**Table 3:** Effect of individual awareness on vaccination intention.

Variables	Frame	Value	N	M (SD)	F	*p*
Understanding of COVID-19	Loss	1	3	2.33(0.577)	9.849	<0.001
		2	10	4.00(0.816)		
		3	27	3.89(0.892)		
		4	86	4.31(0.801)		
		5	17	4.94(0.243)		
Compliance with government COVID-19 control measures		1	1	3.00(0.000)	4.190	0.007
		3	2	2.50(0.707)		
		4	28	4.11(0.737)		
		5	112	4.32(0.862)		
Understanding of COVID-19	Gain	1	1	3.00(0.000)	3.313	0.013
		2	7	3.57(0.976)		
		3	27	3.96(0.898)		
		4	85	4.20(0.737)		
		5	17	4.59(0.712)		
Compliance with government COVID-19 control measures		3	1	3.00(0.000)	8.585	<0.001
		4	38	3.76(0.852)		
		5	98	4.33(0.729)		

Note. The value of “2 (comparative disagreement)” of the loss frame and “1, 2 (very disagreement, comparative disagreement)” of the gain frame that test “Compliance with government COVID-19 control measures” in Table 3 were not chosen by anyone (N = 0), so the statistical results are not displayed.

**Table 4:** Effect of acquaintance vaccination rate on vaccination intention.

Variables	Frame	M (SD)	t	*p*
30%	Loss	3.43(1.166)	12.238	<0.001
	Gain	3.59(1.057)	6.959	<0.001
60%	Loss	3.77(0.899)	29.355	<0.001
	Gain	3.59(1.057)	12.809	<0.001
90%	Loss	4.38(0.919)	10.531	<0.001
	Gain	4.38(0.900)	5.543	<0.001

**Table 5:** Effect of family members’/friends’ vaccination on vaccination intention.

Variables	Frame		N	M (SD)	F	*p*
Family members’/friends’ vaccination	Loss	Yes	77	4.22(0.853)	−0.357	0.722
		No	66	4.27(0.887)		
	Gain	Yes	82	4.35(0.655)	3.566	0.001
		No	55	3.87(0.924)		

**Table 6:** Linear regression analysis of vaccination intention under information framing.

Variables	Frame	M(SD)	Adj.R²	B	t	*p*
**Vaccination Intention**	Loss	4.24(0.866)	0.375	0.616	9.092	<0.001
	Gain	4.16(0.807)	0.300	0.552	7.870	<0.001
**Intention to become vaccinated freely**	Loss	4.37(0.766)	0.582	0.765	14.091	<0.001
	Gain	4.31(0.774)	0.585	0.767	13.871	<0.001

Note. M = mean; SD = standard deviation; B = standardized beta coefficient.

**Table 7:** Effect of risk disclosure on vaccination intention.

Variables.	Frame	M (SD)	t	*p*
Fever, redness, and swelling	Loss	3.27(1.274)	6.368	<0.001
	Gain	2.96(1.084)	6.016	<0.001
Cerebral thrombosis, stroke	Loss	2.40(1.343)	8.893	<0.001
	Gain	2.00(1.043)	8.011	<0.001
Death	Loss	2.04(1.368)	2.468	0.014
	Gain	1.69(1.096)	2.245	0.025
(Not disclosed)	Loss	4.24(0.866)	9.092	<0.001
	Gain	4.16(0.807)	7.870	<0.001

**Table 8:** Effect of perceived vaccine effectiveness on vaccination intention.

Variables	Frame	M (SD)	t	*p*
40%	Loss	3.43(1.166)	10.551	<0.001
	Gain	3.09(1.060)	10.470	<0.001
60%	Loss	3.93(0.924)	15.733	<0.001
	Gain	3.61(1.046)	15.303	<0.001
90%	Loss	4.55(0.854)	6.458	<0.001
	Gain	4.38(0.900)	6.376	<0.001

**Table 9:** Effect of duration of vaccine protection on vaccination intention.

Variables	Frame	M (SD)	t	*p*
Half a year	Loss	3.45(1.137)	9.876	<0.001
	Gain	3.23(1.059)	9.572	<0.001
One year	Loss	3.83(1.035)	19.288	<0.001
	Gain	3.59(1.068)	21.039	<0.001
Two years	Loss	4.17(1.009)	8.388	<0.001
	Gain	4.09(1.014)	8.236	<0.001

## Data Availability

The data that support the findings of this study are available from the corresponding author upon reasonable request.
